# Metabolic Health, Overweight or Obesity, and Lung Function in Older Australian Adults

**DOI:** 10.3390/nu16244256

**Published:** 2024-12-10

**Authors:** Jacob Opio, Katie Wynne, John Attia, Stephen Hancock, Mark McEvoy

**Affiliations:** 1 School of Medicine and Public Health, University of Newcastle, University Drive, Callaghan, NSW 2308, Australia; jacob.opio@uon.edu.au (J.O.); katiejane.wynne@health.nsw.gov.au (K.W.); john.attia@newcastle.edu.au (J.A.); 2 Diabetes and Endocrinology, John Hunter Hospital, New Lambton Heights, NSW 2305, Australia; 3 Hunter Medical Research Institute, School of Medicine and Public Health, University of Newcastle, Callaghan, NSW 2308, Australia; stephen.hancock@newcastle.edu.au; 4 La Trobe Rural Health School, College of Science, Health and Engineering, La Trobe University, Bendigo, VIC 3552, Australia

**Keywords:** obesity, overweight, metabolic health, lung function

## Abstract

**Background:** Few studies have explored the links between adiposity, metabolic health, and lung function. This study examined the cross-sectional association between spirometric lung function and overweight/obesity, with and without metabolic abnormalities, in older adults. **Methods:** The research involved 3,318 older adults from the Hunter Community Study Cohort who had a BMI of 18.5 kg/m^2^ or higher. Participants were grouped based on BMI and metabolic health risk. Obesity was defined as a BMI of 30 kg/m^2^ or more, while metabolic health was determined by the absence of risk factors according to the International Diabetes Federation criteria. Lung function was assessed via spirometry, measuring FEV1, FVC, predicted FEV1, predicted FVC, and FEV1/FVC ratio. Lung dysfunction was classified into restrictive, obstructive, mixed patterns, and deviations from predicted FEV1 and FVC. **Results:** The mean lung function measurements were as follows: FEV1 2.4 L (0.7), FVC 2.9 L (0.8), predicted FEV1% 88.7% (17.6), predicted FVC% 85.6% (15.7), and FEV1/FVC 82.5% (8.5). Compared to the metabolically healthy normal weight (MHNW) group, the odds of lung dysfunction were as follows. For the restrictive pattern, the MHOW group had an odds ratio (OR) of 1.00 (95% CI: 0.70–1.47, *p* = 0.959) and the MHO group had an OR of 1.67 (95% CI: 1.13–2.49, *p* = 0.011). For the obstructive pattern, the MHOW group had an OR of 0.39 (95% CI: 0.20–0.77, *p* = 0.007) and the MHO group had an OR of 0.36 (95% CI: 0.12–1.05, *p* = 0.061). For the mixed pattern, the MHOW group had an OR of 0.39 (95% CI: 0.18–0.87, *p* = 0.021) and the MHO group had an OR of 0.29 (95% CI: 0.10–0.87, *p* = 0.027). **Conclusions:** A higher BMI and variations in metabolic health are associated with an increased likelihood of restrictive lung function patterns. Conversely, obesity is inversely related to obstructive lung function patterns.

## 1. Introduction

The obesity epidemic is a major global health issue with approximately 2 billion people classified as overweight and 650 million classified as having obesity [1,2]. Obesity raises the likelihood of developing metabolic syndrome, type 2 diabetes, and cardiovascular disease [3,4]. Restrictive lung function, which may be present in individuals with conditions such as obesity, leads to mechanical compression of the diaphragm, lungs and chest wall, including a decrease in the compliance of the respiratory system, and is characterised by a reduction in lung volume, which impairs the ability of the lungs to expand fully; while obstructive lung function, which may be present in individuals with conditions such as asthma and chronic obstructive pulmonary disease (COPD), is characterised by airway narrowing or blockage, which impedes the flow of air, especially during expiration [5,6]. Both restrictive and obstructive lung function patterns are associated with chronic respiratory disease (CRD) and non-respiratory diseases including cardiovascular disease and overall mortality [7,8,9]. The 2019 global burden of disease (GBD) study estimated that 454.6 million people suffered from CRD, which was responsible for 103.5 million Disability Adjusted Life Years (DALYs) and 4.0 million deaths, making it the third leading cause of mortality [10]. Risk factors for CRD include environmental or occupational exposures like air pollution and silica, behavioural factors like smoking, and metabolic risk factors such as high Body Mass Index (BMI) and metabolic syndrome [10]. The impact of overweight and obesity on lung function is being studied. These conditions are characterised by excess body fat and can be assessed using various measures. Body Mass Index (BMI) is one method, with overweight defined as a BMI between 25 and 29.9 kg/m^2^, and obesity as a BMI of 30 kg/m^2^ or higher. Another approach is percentage body fat, where obesity in women is considered ≥35% and overweight between 30 and 35%, while for men, obesity is ≥30% and overweight between 25 and 29%. Abdominal obesity is also assessed, with a waist circumference of ≥88 cm for women and ≥102 cm for men indicating this condition [11,12]. Overweight and obesity are linked to metabolic diseases, including metabolic syndrome. While metabolic syndrome is not classified as a disease in itself, it refers to a group of risk factors that increase the likelihood of developing atherosclerosis [11]. The diagnostic criteria for metabolic syndrome are described in Table 1.

Multiple studies have demonstrated an inverse association between overweight, obesity, and lung function. Systematic reviews and meta-analyses conducted between 2012 and 2021 consistently reported overweight or obesity to be associated with reduced lung function, as measured by forced expiratory volume in one second (FEV1) and forced vital capacity (FVC) [13,14,15]. Other studies also reported declines in FEV1 and FVC linked to these conditions [16,17,18,19,20]. Notably, the impact of overweight or obesity on lung function varies by gender [21].

Several studies have reported that metabolic syndrome is associated with lung function [22,23,24,25]. A recent meta-analysis of fifteen studies, including 10,285 cases and 25,416 controls, found significant reductions in FVC (pooled standardised mean difference (SMD) = −0.247, 95%CI: −0.327 to −0.2167, *p* < 0.001) and FEV1 (pooled SMD = −0.205, 95%CI: −0.3278 to –0.133, *p* < 0.001) among individuals with metabolic syndrome [26]. Overweight, obesity, and metabolic syndrome are associated with reduced lung function, but there is a subgroup called metabolically healthy obesity (MHO) that shows little or no metabolic dysfunction. This group makes up about 10 to 40% of individuals with obesity [27,28,29]. Research on MHO and lung function is limited. A longitudinal study by Song et al. (2023) involving 253,698 Korean adults found that both MHO and metabolically unhealthy obesity (MUHO) were associated with a restrictive lung function pattern, with a stronger association in the MUHO group (HR = 1.38) than in the MHO group (HR = 1.15). Obesity was inversely associated with an obstructive lung function pattern, with a greater decline in FVC than FEV1 [30]. Another cross-sectional study by J Lee et al. (2022), involving 114,143 Korean adults, found that MHO participants had higher FEV and FVC, but the lowest FEV1/FVC ratio (*p* < 0.001). Compared to metabolically healthy normal weight (MHNW) individuals, the odds ratios (ORs) for FEV1 < 80% were as follows: for MHO = 0.871, for metabolically unhealthy but no obesity (MUNO) = 1.274, and for MUHO = 1.18. For FVC < 80%, the ORs were as follows: MHO = 0.70, MUNO = 1.24, and MUHO = 1.22. There were no significant differences in the OR for FEV1/FVC < 0.7 among these groups [31]. An earlier cross-sectional study by HY Lee et al. (2019) involving 10,071 Korean adults, found that metabolically unhealthy individuals were more likely to have reduced lung function compared to metabolically healthy participants, regardless of obesity status [32]. Limited research has explored the connections between metabolic health, overweight, obesity, and lung function or dysfunction, and no studies have been published from Australia. There is increasing interest in individuals who are classified as having overweight or obesity but do not present any cardiometabolic abnormalities. However, the evidence regarding the relationship between MHO and lung function is inconsistent. Considering the high prevalence of overweight/obesity and metabolic dysfunction among older adults, this study seeks to examine the link between these conditions and lung function in older Australian adults.

## 2. Methods

### 2.1. Sample

**Figure 1:**
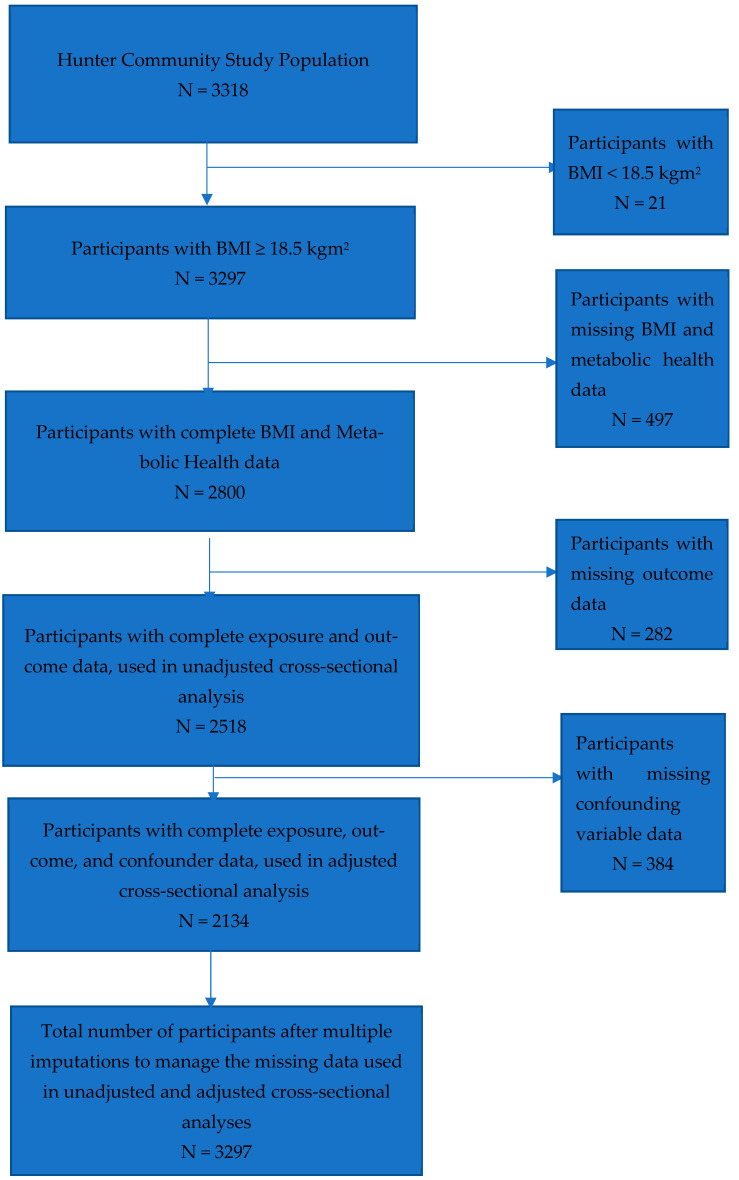
Flow diagram for the selection of participants from the Hunter Community Study with complete data, for six obesity phenotypes and confounders after the exclusion of participants with BMI < 18.5 kgm^2^, missing BMI/metabolic health data, or missing outcome and confounder data, and multiple imputations to manage the missing data.

This study presents findings based on a secondary analysis of baseline data from the Hunter Community Study (HCS) (refer to Figure 1). The HCS was a population-based cohort study targeting men and women aged 55–85 years residing in Newcastle, New South Wales (NSW), Australia. Detailed methodologies of the study have been previously outlined [33]. The study involved 3318 individuals who completed baseline surveys, yielding a response rate of 44.5%. While the gender distribution was similar between respondents and non-respondents, the respondents were generally younger. The demographic characteristics of the HCS participants were comparable to those of the Hunter region, NSW, and the broader Australian population in terms of gender and marital status, though the cohort was somewhat younger than the national and state averages.

For the current analysis, participants classified as underweight (BMI < 18.5 kg/m^2^) were excluded, as the focus was on comparing adults with overweight or obesity to those of normal weight. This research adhered to the ethical standards outlined in the Declaration of Helsinki. All participants gave informed consent, and the study procedures were approved by the University of Newcastle Human Research Ethics Committee (approval code: H-820-0504).

### 2.2. Exposure Measurement

Individuals considered metabolically healthy were those who did not exhibit any of the following risk factors: triglyceride levels of 1.7 mmol/L or higher, HDL cholesterol levels below 1.0 mmol/L in men or below 1.3 mmol/L in women, or use of lipid-lowering drugs; blood pressure readings of 130/85 mmHg or higher, or use of antihypertensive medications; and fasting serum glucose levels of 5.6 mmol/L or higher, or self-reported diabetes as defined by the IDF criteria [34]. BMI was classified according to the World Health Organization (WHO) standards into normal weight (18.5–24.9 kg/m^2^), overweight (25–29.9 kg/m^2^), or obesity (≥30.0 kg/m^2^) [35]. These BMI categories were combined with metabolic health status (metabolically healthy or unhealthy) to create six distinct groups: MHNW, MHOW, MHO, MUNW, MUOW, and MUO.

Waist-to-Hip Ratio (WHR) was categorised using WHO cut-offs: high risk if the WHR was ≥0.90 cm for men and ≥0.85 cm for women, and low risk if it was below these values [36]. WHR and metabolic health were combined to form four WHR–metabolic health groups: metabolically healthy low risk, metabolically heathy high risk, metabolically unhealthy low risk and metabolically unhealthy high risk.

### 2.3. Outcome Measurement

#### 2.3.1. Primary Outcomes

Lung function was measured using spirometry, according to the American Thoracic Society (ATS) guidelines [37], using electronic spirometers (Micro Medical SpiroUSB, Cardinal Health, Kent, UK) with Spida 5 software (Carefusion Ltd., Kent, UK) and predicted values of Gore [38]. The five key lung function measures were FEV1, FVC, FEV1/FVC, %Predicted FEV1, and %Predicted FVC. Lung dysfunction was defined as follows: restrictive lung function pattern (RP) (FVC < 80% and FEV1/FVC ≥ 0.7), obstructive lung function pattern (OP) (FVC > 80% and FEV1/FVC < 0.7), and mixed lung function pattern (MP) (FVC < 80% and FEV1/FVC < 0.7). Spirometers were calibrated daily using a 3 L syringe. All the values obtained were corrected to body temperature and ambient pressure, and saturated with water vapour (BTPS), with an assumed fixed room temperature and atmospheric pressure.

#### 2.3.2. Measurement of Potentially Confounding Variables

**Figure 2:**
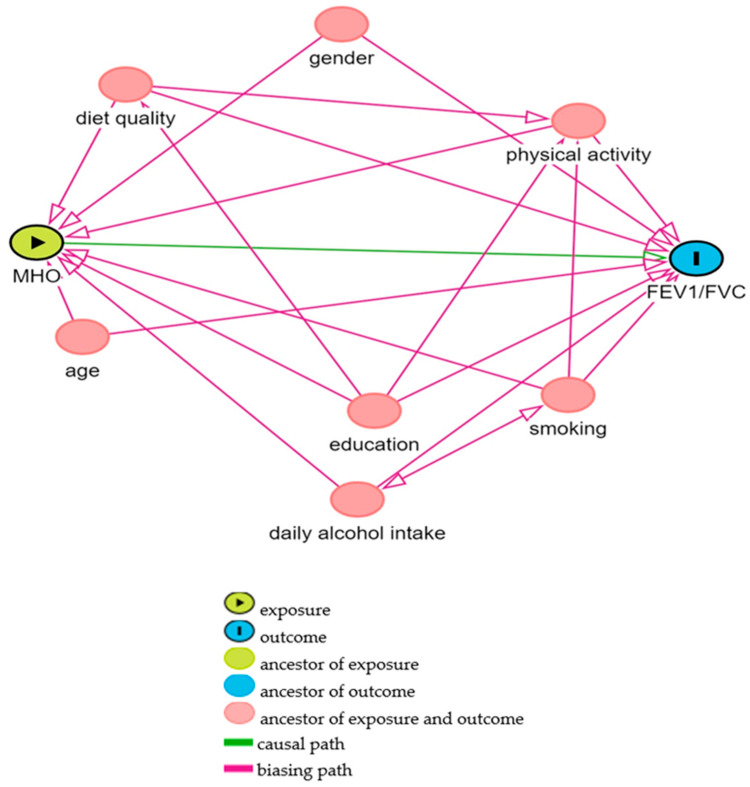
Directed acyclic graph (DAG) of potential confounding variables for the effect of metabolic health–BMI status on lung function. Exposure: metabolically healthy obesity (MHO). Outcome: lung function (FEV1, FVC, FEV1/FVC). Minimal sufficient adjustment set included age, gender, physical activity, education, diet quality, alcohol intake, and smoking.

To analyse the association between MHOW or MHO and lung function, directed acyclic graphs (DAGs) [39] were used to identify confounding variables, guided by expert knowledge and scientific literature. The DAGs indicated that age, gender, education, smoking, alcohol intake, diet quality, and physical activity needed to be controlled (see Figure 2). Age, gender, and education were obtained from self-administered questionnaires. Details on the coding of each variable are described in Table 1. Alcohol consumption was reported as the mean number of standard alcoholic drinks per day, as per the Australian NHMRC guidelines [40]. Diet quality was assessed using the Australian Recommended Food Score (ARFS), calculated from a validated 145-item Food Frequency Questionnaire (FFQ). The ARFS evaluates diet based on the variety of foods within categories defined by the Australian Dietary Guidelines, with a maximum score of 74, where a higher score indicates better diet quality [41,42,43,44,45].

#### 2.3.3. Study Design and Statistical Analysis

This study investigated the relationships between BMI, WHR, and BMI/WHR–metabolic health groups with lung function and lung dysfunction patterns. Participants were divided into six BMI–metabolic health groups and four WHR–metabolic health groups.

Baseline characteristics were compared using Chi-square tests and ANOVA with Bonferroni adjustments, considering *p* < 0.05 as significant. Multiple logistic regression estimated the odds ratios (ORs) and 95% confidence intervals (CIs) for lung dysfunction patterns, using MHNW as the reference group. Multiple imputation created twenty datasets to address missing data, assuming they were missing at random. Both complete-case and imputed data analyses are presented.

Multiple linear regression assessed the association between the BMI–metabolic health groups and lung function, with assumptions verified. An interaction analysis examined whether the odds of decreased lung function varied by BMI–metabolic health group combination, using interaction terms in the logistic regression model. The Relative Excess Risk due to Interaction (RERI) and its 95% CI were calculated.

Gender differences in the odds of decreased lung function were assessed with interaction terms for ‘gender × BMI–metabolic health group’. Metabolically healthy normal weight females were the reference group. ORs and 95% CIs were estimated, with *p* < 0.05 considered significant.

A sensitivity analysis, conducted separately by gender due to different WHR risk definitions, assessed the association between WHR–metabolic health groups and lung function. The low-risk WHR–metabolically healthy group served as the reference. All model assumptions were verified.

## 3. Results

Out of the 3318 participants in the HCS cohort, 21 were removed due to a BMI below 18.5 kg/m^2^. This left 3297 participants with a BMI of 18.5 kg/m^2^ or above. Among them, 497 had incomplete BMI and metabolic health information, which left 2800 participants with complete data. After removing 282 participants with incomplete outcome data, 2518 participants remained for the unadjusted complete-case analysis. For the adjusted analysis, 2134 participants were included, after accounting for 384 participants with missing data on confounding variables. Imputation of missing data for 1163 participants led to a final dataset of 3297 for the analysis using imputed data (see Figure 1).

### 3.1. Baseline Characteristics of Study Population

The baseline characteristics of the study population, categorised according to BMI–metabolic health group, are presented in Table 2. For the overall sample, the means (SDs) for FEV1, FVC, predicted FEV1%, and predicted FVC% were 2.4 (0.7), 2.9 (0.8), 88.7% (17.6), and 82.5% (8.5), respectively. The proportion of participants with lung dysfunction were as follows: restricted pattern—32.0% (801/2518), obstructive pattern—4.0% (93/2518), and mixed pattern—3.0% (85/2518).

Approximately 21.1% (531/2518) and 11.1% (280/2518) of the total population were classified as MHOW and MHO, respectively. Obesity was present in about 34.4% (866/2518) of the sample, with 32.3% (280/866) of this group categorised as MHO. The average age of the participants was 66.2 years (SD 7.5). Metabolically healthy individuals were generally younger than those categorised as metabolically unhealthy. Slightly more than half of the sample (i.e., 57.2%, 1441/2518) were female.

### 3.2. BMI–Metabolic Health Group and Odds of Lung Function/Dysfunction

For the primary analysis, only the findings from the imputed dataset are presented, as these provide a more valid estimate of effects [46,47,48]. The sensitivity analysis results, based on the association between WHR, metabolic health, and lung function, are presented in Supplementary Table S1.

The adjusted models (see Table 3) and findings across all outcomes are summarised in Table 4.

### 3.3. Restrictive Pattern

For the restrictive lung function pattern, increased ORs were observed in MHO (OR = 1.67, 95% CI: 1.13–2.49, *p* = 0.011) and MUO (OR = 2.23, 95% CI: 1.53–3.25, *p* = 0.000). In MUNW (OR = 1.23, 95% CI: 0.81–1.86, *p* = 0.333) and MUOW (OR = 1.37, 95% CI: 0.96–1.95, *p* = 0.081), the increases were not significant. The restrictive pattern in MHOW was similar to MHNW (OR = 1.00, 95% CI: 0.70–1.47, *p* = 0.959).

### 3.4. Obstructive Pattern

Compared with the MHNW group, the adjusted ORs for an obstructive lung function pattern were as follows: 0.39 (95% CI: 0.20–0.77, *p* = 0.007) for MHOW and 0.36 (95% CI: 0.12–1.05, *p* = 0.061) for MHO. In MUNW, the OR was 0.73 (95% CI: 0.29–1.88, *p* = 0.510); in MUOW, it was 0.50 (95% CI: 0.25–1.02, *p* = 0.055); and in MUO, it was 0.33 (95% CI: 0.14–0.77, *p* = 0.011).

### 3.5. Mixed Pattern

For the mixed lung function pattern, the ORs were 0.39 (95% CI: 0.18–0.87, *p* = 0.021) for MHOW, 0.29 (95% CI: 0.10–0.87, *p* = 0.027) for MHO, 1.15 (95% CI: 0.44–2.97, *p* = 0.771) for MUNW, 0.37 (95% CI: 0.17–0.82, *p* = 0.015) for MUOW, and 0.52 (95% CI: 0.24–1.15, *p* = 0.105) for MUO.

### 3.6. FEV1 to Predicted FEV1 < 80%

Regarding reduced lung function according to FEV1 to predicted FEV1 < 80%, the ORs significantly increased in MUNW (OR = 1.66, 95% CI: 1.05–2.61, *p* = 0.029) and MUO (OR = 1.67, 95% CI: 1.15–2.43, *p* = 0.008). The ORs were increased but not significant in MHO (OR = 1.17, 95% CI: 0.75–1.80, *p* = 0.487) and MUOW (OR = 1.27, 95% CI: 0.88–1.83, *p* = 0.205), while it was not increased in MHOW (OR = 0.86, 95% CI: 0.58–1.27, *p* = 0.443).

### 3.7. FVC to Predicted FVC < 80%

For reduced lung function according to FVC to predicted FVC < 80%, significant increases were found in MHO (OR = 1.56, 95% CI: 1.07–2.28, *p* = 0.021) and MUO (OR = 2.05, 95% CI: 1.48–2.84, *p* = 0.000). The increases were not significant in MUNW (OR = 1.14, 95% CI: 0.76–1.71, *p* = 0.534) and MUOW (OR = 1.25, 95% CI: 0.91–1.73, *p* = 0.172), and there was no increase in MHOW (OR = 0.95, 95% CI: 0.68–1.34, *p* = 0.785).

### 3.8. BMI Category (Normal Weight, Overweight, and Obesity) with Normal Weight as Reference Group

Compared to normal weight, the odds of an obstructive lung function pattern were lower in both participants classified as overweight (OR = 0.53, 95% CI: 0.30–0.92, *p* = 0.026) and obese (OR = 0.33, 95% CI: 0.17–0.64, *p* = 0.001). Similarly, the OR for a mixed lung function pattern was reduced in those with overweight (OR = 0.35, 95% CI: 0.20–0.59, *p* < 0.001) and obesity (OR = 0.41, 95% CI: 0.22–0.76, *p* = 0.006). However, the odds for a restrictive lung function pattern were increased in overweight individuals (OR = 1.12, 95% CI: 0.88–1.42, *p* = 0.347) and individuals with obesity (OR = 1.85, 95% CI: 1.42–2.41, *p* < 0.001).

Regarding reduced lung function according to FEV1 to predicted FEV1 < 80%, the OR was increased in those with obesity (OR = 1.20, 95% CI: 0.92–1.57, *p* = 0.187), but not in overweight individuals (OR = 0.80, 95% CI: 0.57–1.11, *p* = 0.185). For reduced lung function according to FVC to predicted FVC < 80%, the OR was increased in those with obesity (OR = 1.74, 95% CI: 1.36–2.22, *p* < 0.001) but not in overweight individuals (OR = 1.00, 95% CI: 0.81–1.25, *p* = 0.966) (see Table 2).

Given that BMI has limitations, we carried out a sensitivity analysis using WHR for consistency. The odds ratios were similar and pointed in the same direction as for BMI (see Supplementary Table S1).

### 3.9. Reduced Lung Function According to FVC, FEV1, Predicted FVC, and Predicted FEV1

The coefficient of FVC, FEV1, predicted FVC, and predicted FEV1 showed a reduction in lung function with an increase in BMI (see Supplementary Table S12). A similar association was observed between WHR and reduced lung function, according to WHR from low to high risk (see Supplementary Table S13).

### 3.10. Odds of Lung Dysfunction Pattern by Interaction Between BMI and Metabolic Health Category

We examined how the interaction between BMI and metabolic health was associated with the odds of obstructive, restrictive, and mixed lung dysfunction patterns. The analysis revealed non-significant interactions for both overweight and obesity with metabolic unhealthy across different metrics (RERI values ranging from −1.18 to 0.33). Overall, none of the RERI values reached statistical significance, indicating a lack of strong evidence for positive or negative interactions.

See Supplementary Information (Supplementary Tables S2–S6 and Section 3).

### 3.11. Association Between BMI–Metabolic Health Group and Reduced Lung Function According to Gender

In an adjusted analysis, using females categorised with MHNW as the reference group, the odds of reduced lung function in males were different and higher than in the corresponding females in all BMI–metabolic health groups for all outcomes (i.e., restrictive pattern, obstructive pattern, mixed pattern, FEV1 to predicted FEV1, and FVC to predicted FVC) (see Supplementary Tables S7–S11).

### 3.12. Within-Strata Effect of Gender on Odds of Reduced Lung Function

The odds of reduced lung function in each gender, with the reference group being MHNW for the respective gender, are also presented in Supplementary Tables S7–S11. Compared with the male MHNW group, the odds of a restrictive lung function pattern were positively associated with BMI within the male strata, but inversely associated with BMI in the obstructive pattern. A similar trend applied to the odds of restrictive patterns and obstructive patterns of lung function in the female strata with MHNW females as a reference group. The odds of reduced lung function for mixed pattern, FEV1 to predicted FEV1, and FVC to predicted FVC in both the male and female strata showed a mixed picture.

## 4. Discussion

This study investigated the association between overweight/obesity, metabolic health, and various patterns of lung function and reduced lung function, as measured by FEV1 and FVC, in older adults. The findings reveal significant associations that contribute to an understanding of how metabolic health and body composition may impact lung function.

### 4.1. Restrictive Lung Function Pattern

The association between metabolic health and restrictive lung function patterns revealed that MHO and MUO categories had increased odds of a restrictive pattern, with ORs of 1.67 and 2.23, respectively. This finding aligns with the existing literature suggesting that restrictive lung function patterns are more pronounced in individuals with metabolic dysfunction, particularly when combined with obesity. In contrast, the increase in restrictive patterns among MUNW and MUOW was not significant, and MHOW did not differ from MHNW, highlighting the variability in restrictive lung function across different categories of metabolic health status.

### 4.2. Obstructive Lung Function Pattern

This analysis indicates that metabolically healthy individuals with overweight (MHOW) and those who are metabolically healthy with obesity (MHO) show lower odds of an obstructive lung function pattern, compared to metabolically healthy normal weight (MHNW) individuals. Specifically, MHOW (OR = 0.39) and MHO (OR = 0.36) have substantially lower odds of exhibiting an obstructive pattern, suggesting a protective effect of metabolic health against obstructive lung conditions [49]. However, the results for MHO approached but did not reach statistical significance (*p* = 0.061), indicating a potential trend that warrants further investigation.

Conversely, metabolically unhealthy individuals with obesity (MUO) showed a significantly reduced odds ratio for obstructive lung function (OR = 0.33). This may indicate that while metabolic health generally offers protection, the impact of obesity on obstructive patterns can be significant. The non-significant results for other categories of metabolically unhealthy without obesity (MUNW, MUOW) suggest a more complex relationship that could be influenced by additional factors, such as the severity of obesity or concurrent health conditions. The complex relationship between obesity and respiratory conditions was demonstrated in the SUMMIT trial, where mortality in participants with moderate COPD and increased cardiovascular risk was reduced in those with obesity, but the protective nature did not persist beyond a BMI of 40 kgm^2^ [50].

### 4.3. Mixed Lung Function Pattern

For mixed lung function patterns, MHOW and MHO exhibited reduced odds (OR = 0.39 and OR = 0.29, respectively), which might suggest that metabolic health provides some level of protection against mixed patterns of lung dysfunction. Conversely, MUOW showed a lower odds ratio (OR = 0.37), but the results for MUO (OR = 0.52) and MUNW (OR = 1.15) did not show clear patterns. This mixed picture suggests that the relationship between metabolic health, obesity, and mixed lung function patterns is multifaceted and potentially influenced by factors such as the interplay between obstructive and restrictive components.

### 4.4. Reduced Lung Function: FEV1 and FVC

Regarding reduced lung function, these results demonstrate increased odds ratios for FEV1 < 80% in MUNW (OR = 1.66) and MUO (OR = 1.67), indicating that metabolic dysfunction, particularly with obesity, is associated with a higher likelihood of reduced FEV1. Similarly, for FVC < 80%, significant increases were observed in MHO (OR = 1.56) and MUO (OR = 2.05). This suggests that while metabolic health may mitigate some of the negative effects of obesity on lung function, the presence of obesity may still be associated with a significant risk of reduced lung function.

### 4.5. BMI and Lung Function Patterns

The analysis comparing BMI categories reveals that both overweight and obesity are associated with decreased odds of obstructive and mixed lung function patterns compared to normal weight. However, obesity is linked to increased odds of restrictive lung function. This finding emphasises that while obesity may be protective against some lung function patterns, it may be associated with a significantly higher risk of restrictive patterns.

### 4.6. Sensitivity Analysis with WHR

The sensitivity analysis using Waist-to-Hip Ratio (WHR) corroborated the BMI findings, suggesting that the observed associations with lung function patterns and reduced lung function are consistent across different measures of body composition. This strengthens the robustness of these findings, and underscores the importance of considering both BMI and WHR in assessing lung function risks.

### 4.7. Gender Differences

These findings highlight notable gender differences in the odds of reduced lung function. Men generally exhibit higher odds of restrictive patterns compared to women across all BMI and metabolic health groups. In contrast, the relationship with obstructive patterns varies by gender, with different trends observed in males and females. These results underscore the need for gender-specific considerations in understanding and addressing lung function impairments.

### 4.8. Comparisons with Other Studies

These findings align with a longitudinal study of 253,698 Korean adults, linking obesity and metabolic health with a restrictive lung function pattern, but finding an inverse association with an obstructive lung function pattern [30]. This contrasts with earlier cross-sectional studies which associated reduced lung function with metabolic health abnormalities, not obesity [31,32,49]. This study defined metabolically healthy more strictly (no risk factors), potentially reducing misclassification. Lung dysfunction correlates with a number of metabolic risk factors [51,52,53,54]. Additionally, we categorised obesity into normal weight, overweight, and obesity, creating six distinct obesity–metabolic health phenotypes, unlike studies combining normal weight and overweight.

Previous research has reported that obesity leads to a restrictive lung function pattern by limiting lung expansion and decreasing lung volume, due to effects on the diaphragm and chest wall compliance [7,55,56,57,58,59]. The association between restrictive lung function and metabolic health may involve insulin resistance [51,60]. The inverse association between obesity and the FEV1/FVC ratio may arise because obesity affects FVC more than FEV1, misleadingly suggesting a positive correlation between obesity and lung function. Consequently, screening spirometry might miss functional airway issues in healthy individuals, as a FEV1/FVC ratio below 0.7 typically indicates significant airway obstruction [61,62]. Additionally, obesity is also associated with pro-inflammatory cytokines and lower levels of adiponectin [63,64,65]. In high-income countries, low-BMI-related inflammation might protect against lung function decline [64]. Central obesity is a significant driver of lung function impairment [66,67]. The WHO recommend WHR as a better indicator of body fat distribution and cardiovascular risk than waist circumference and BMI [36,68,69,70]. High WHR values, reflecting increased visceral fat, may contribute to lung function decline even in metabolically healthy normal weight.

Finally, males had greater declines in lung function across all BMI–metabolic health categories compared to females, which is consistent with previous studies showing greater reductions in FEV1 and FVC in males [71] (see Supplementary Tables S7–S11).

### 4.9. Strength and Limitations

This research is the first to explore the relationship between BMI–metabolic health phenotypes and lung function among adults in Australia. A major strength is the use of validated, objective measures for assessing lung function. By classifying BMI and metabolic health into six distinct groups, misclassification bias was reduced. The study sample represents community-dwelling older Australians, enhancing the generalisability of the findings.

However, limitations include a relatively small sample size within each BMI–metabolic health category, which may have affected the precision of effect estimates and statistical significance. This is likely due to the community-dwelling study population, which was slightly younger than the national and state population, with few participants with symptomatic lung disease. Despite this, the observed effect sizes are informative, and integrating these results with other studies (i.e., meta-analysis) could refine the estimates. The cross-sectional design limits the ability to draw conclusions about the temporal relationships between BMI–metabolic health phenotypes and a decline in lung function. In the future, longitudinal studies are necessary to examine changes in BMI, inflammatory markers, and metabolic health over time, and to investigate how these changes affect lung function.

## 5. Conclusions

This study highlights that an increase in BMI and variations in metabolic health status are associated with an increased likelihood of a restrictive lung function pattern. Specifically, participants classified with obesity exhibited the highest odds of a restrictive lung function pattern across all metabolic health statuses. Furthermore, metabolically unhealthy individuals had higher odds of a restrictive lung function pattern compared to those who were metabolically healthy.

Conversely, obesity was found to be inversely associated with an obstructive lung function pattern. The study also identified that males experienced a greater reduction in lung function compared to females across all BMI–metabolic health phenotypes.

## Figures and Tables

**Table 1:** Diagnostic Criteria for Metabolic Syndrome.

Cardiometabolic Risk Factors	Presence of Three or More Risk Factors Constitutes Metabolic Syndrome
Abdominal obesity	waist circumference ≥ 102 cm in males, ≥88 cm in females
Triglycerides	≥1.7 mmol/L
High Density Lipoprotein cholesterol (HDL)	<1.04mmol/L in males, <1.30 mmol/L in females
Blood pressure	≥130/85 mmHg
Fasting glucose	≥5.6 mmol/L

**Table 2:** Characteristics of study population according to BMI categories and metabolic health groups.

Characteristics		Metabolically Healthy			Metabolically Unhealthy			Overall
		Normal Weight	Overweight	Obesity	Normal Weight	Overweight	Obesity	
Percentage and number of participants		12.3%	12.1%	11.1%	8.1%	24.1%	23.3%	100.0%
		(311)	(531)	(280)	(203)	(607)	(586)	(2518)
Number of participants with normal lung function		211	362	166	130	374	296	1539
Percentage and number of participants with lung dysfunction	Combined total	100	169	114	73	233	290	979
	Restrictive pattern	20.0%	25.0%	37.0%	26.0%	31.0%	44.0%	32.0%
		(63)	(133)	(102)	(52)	(193)	(258)	(801)
	Obstructive pattern	7.0%	4.0%	2.0%	5.0%	4.0%	2.0%	4.0%
		(20)	(19)	(6)	(10)	(25)	(13)	(93)
	Mixed pattern	5.0%	3.0%	2.0%	5.0%	3.0%	3.0%	3.0%
		(17)	(17)	(6)	(11)	(15)	(19)	(85)
Age (mean SD) (median, IQR)		65.3 (7.9)	64.8 (7.0)	63.8 (6.6)	68.9 (7.7)	67.9 (7.7)	66.5 (7.0)	66.2 (7.5)
		63.1 (11.3)	63.1 (10.2)	62.0 (9.1)	68.6 (11.4)	66.9 (12.8)	65.6 (10.9)	64.8 (11.7)
Female gender (%, N)		70.7%	50.8%	63.6%	71.9%	48.9%	56.3%	57.2%
		(220)	(270)	(178)	(146)	(297)	(330)	(1441)
Current smoker (%, N)		9.3%	8.6%	8.3%	8.5%	4.8%	7.0%	7.4%
		(32)	(51)	(26)	(19)	(32)	(44)	(204)
Ever smoker (%, N)		31.0%	37.5%	39.3%	32.7%	42.2%	39.6%	38.1%
		(107)	(223)	(123)	(73)	(279)	(250)	(1055)
Never smoker (%, N)		59.7%	54.0%	52.4%	58.7%	53.0%	53.5%	54.5%
		(206)	(321)	(164)	(131)	(350)	(338)	(1510)
BMI (mean, SD)		23.0	27.3	33.4	23.1	27.6	34.4	28.8
		(1.5)	(1.4)	(3.6)	(1.5)	(1.4)	(4.2)	(4.9)
Highest level of education: primary or secondary (%, N)		45.0%	39.8%	46.5%	47.1%	47.7%	53.3%	46.7%
		(157)	(236)	(146)	(105)	(317)	(335)	1296
Highest level of education: university or tertiary-level (%, N)		55.0%	60.2%	53.5%	52.9%	52.3%	46.7%	53.3%
		(192)	(357)	(168)	(118)	(348)	(294)	(1477)
Alcohol intake (mean, SD)		1.8 (2.1)	2.1 (2.5)	2.1 (2.7)	1.3 (2.1)	2.2 (3.6)	2.0 (2.8)	2.0 (2.8)
Physical activity (mean, SD)		8377.9	7285.1	6274.2	7258.7	6802.0	5580.9	6815.6
		(2975.1)	(3100.1)	(2835.8)	(3092.5)	(3238.2)	(2951.4)	(3174.2)
Recommended Food Score (mean, SD)		28.3	28.6	27.9	28.2	27.8	27.8	28.1
		(8.1)	(8.1)	(7.6)	(8.4)	(8.1)	(7.8)	(8.0)
Metabolic parameters								
Diastolic BP (mmHg) (mean, SD)		76.4	79.8	79.9	76.7	79.1	78.3	78.6
		(9.6)	(9.5)	(9.3)	(9.5)	(10.1)	(10.5)	(9.9)
Systolic BP (mmHg) (mean, SD)		129.7	134.3	133.7	137.5	139.5	136.8	135.7
		(18.6)	(18.1)	(16.7)	(20.3)	(19.6)	(17.4)	(18.7)
HDL-C (mmol/l) (mean, SD)		1.6	1.4	1.4	1.5	1.3	1.2	1.4
		(0.4)	(0.4)	(0.4)	(0.4)	(0.3)	(0.3)	(0.4)
Triglycerides (mmol/l) (mean, SD)		1.0	1.2	1.4	1.2	1.5	1.8	1.4
		(0.4)	(0.6)	(0.9)	(0.6)	(0.9)	(1.4)	(1.0)
Glucose (mmol/l) (mean, SD)		4.7	4.8	4.9	4.9	5.3	5.7	5.1
		(0.6)	(0.6)	(0.6)	(1.0)	(1.4)	(1.6)	(1.2)
Lung function								
FEV_1_ (L) (mean, SD)		2.4	2.6	2.4	2.2	2.4	2.3	2.4
		(0.7)	(0.7)	(0.7)	(0.7)	(0.7)	(0.7)	(0.7)
FEV_1_ (% pred) (mean, SD)		90.7	91.0	89.0	87.4	88.9	85.6	88.7
		(16.6)	(15.3)	(19.1)	(19.1)	(16.7)	(19.1)	(17.6)
FVC (L) (mean, SD)		3.0	3.2	2.9	2.7	3.0	2.7	2.9
		(0.8)	(0.9)	(0.8)	(0.8)	(0.8)	(0.8)	(0.8)
FVC (% pred) (mean, SD)		89.5	87.5	83.5	86.8	85.9	81.9	85.6
		(15.2)	(13.7)	(13.7)	(16.9)	(15.0)	(17.8)	(15.7)
FEV_1_/FVC (mean, SD)		80.8	82.5	83.8	80.7	82.6	83.4	82.5
		(9.4)	(7.7)	(8.5)	(9.7)	(8.3)	(8.0)	(8.5)

Normal weight: BMI = 18.5 kgm^2^–24.9 kgm^2^; overweight: BMI = 25 kgm^2^–29.9 kgm^2^; obesity: BMI ≥ 30 kgm^2^. Metabolically healthy: presence of no metabolic risk factors; metabolically unhealthy: presence of one or more metabolic risk factors. Metabolically healthy normal weight (MHNW). Metabolically healthy overweight (MHOW). Metabolically healthy obesity (MHO). Metabolically unhealthy normal weight (MUNW). Metabolically unhealthy overweight (MUOW). Metabolically unhealthy obesity (MUO). Categorical variables are presented as percentages (%). SD = Standard deviation. BMI = Body Mass Index (kg/m^2^). SBP = Systolic Blood Pressure (mmHg). DBP = Diastolic Blood Pressure (mmHg). Trig = Triglycerides (mm/L). HDL-C = high-density lipoprotein cholesterol (mm/L). Fasting glucose (mmol/L). Education was classified according to the highest level of education attained, as primary and secondary schooling completed, or trade qualification, university, and other tertiary-level study completed. Physical activity was measured by recorded step counts using a pedometer worn by participants for seven consecutive days during waking hours, and was reported as the mean number of steps per day. Smoking frequency was measured by the number of cigarettes per day and reported as never, ever, or current smoker. Alcohol consumption was measured by the number of standard drinks per day, defined according to the Australian National Health and Medical Research Council (NHMRC) guidelines, and reported as the mean number of standard alcohol drinks per day. FEV1 = forced expiratory volume in 1 s (L). FVC = forced vital capacity (L). FEV1/FVC (%).

**Table 3:** Cross-sectional associations of BMI–metabolic health group and BMI category with lung function and lung dysfunction pattern, with multiple imputation of missing data.

Obesity Measures		Metabolically Healthy			Metabolically Unhealthy			BMI Category		
Body Mass Index (BMI)		18.5–24.9	25–29.9	≥30	18.5–24.9	25–29.9	≥30	18.5–24.9	25–29.9	≥30
Percentage and number of participants in complete-case analysis		12.3%	12.1	11.1	8.1%	24.1%	23.3%	20.4%	45.2%	34.4%
		(311)	(531)	(280)	(203)	(607)	(586)	(528)	(1169)	(892)
Percentage and number of participants with lung dysfunction	Restrictive pattern	23.0%	26.9%	38.1%	28.6%	34.0%	46.6%	25.2%	30.8%	43.6%
		(63)	(133)	(102)	(52)	(193)	(258)	(118)	(336)	(370)
	Obstructive pattern	8.7%	5.0%	3.5%	7.1%	6.3%	4.2%	8.1%	5.7%	3.8%
		(20)	(19)	(6)	(10)	(25)	(13)	(31)	(46)	(19)
	Mixed pattern	7.5%	4.5%	3.5%	7.8%	3.9%	6.0%	7.7%	4.1%	5.0%
		(17)	(17)	(6)	(11)	(15)	(19)	(29)	(32)	(25)
	FEV1 to predicted FEV1 < 80%	19.7%	20.0%	25.5%	32.0%	28.2%	35.0%	24.3%	24.5%	31.8%
		(61)	(106)	(71)	(65)	(171)	(205)	(128)	(286)	(283)
	FVC to predicted FVC < 80%	25.8%	28.3%	38.7%	31.0%	34.3%	47.3%	27.9%	31.5%	44.3%
		(80)	(150)	(108)	(63)	(208)	(277)	(147)	(368)	(395)
Obstructive pattern FEV1/FVC < 0.7 and predicted FVC ≥ 80%. (OR, 95% CI, *p*-value)	Total	1	0.39	0.36	0.73	0.50	0.33	1	0.53	0.33
			0.20–0.77	0.12–1.05	0.29–1.88	0.25–1.02	0.14–0.77		0.30–0.92	0.17–0.64
			0.007	0.061	0.510	0.055	0.011		0.026	0.001
	Female	1	0.37	0.30	0.51	0.31	0.26	1	0.48	0.33
			0.15–0.89	0.09–0.99	0.14–1.81	0.11–0.85	0.09–0.74		0.23–1.02	0.13–0.82
			0.026	0.049	0.293	0.023	0.013		0.058	0.017
	Male	1	0.48	0.44	1.06	0.71	0.43	1	0.56	0.34
			0.19–1.21	0.09–2.00	0.32–3.51	0.28–1.80	0.15–1.29		0.28–1.14	0.15–0.74
			0.119	0.278	0.925	0.464	0.129		0.108	0.008
Restrictive pattern FEV1/FVC ≥ 0.7 and predicted FVC < 80%. (OR, 95% CI, *p*-value)	Total	1	1.00	1.67	1.23	1.37	2.23	1	1.12	1.85
			0.70–1.47	1.13–2.49	0.81–1.86	0.96–1.95	1.53–3.25		0.88–1.42	1.42–2.41
			0.959	0.011	0.333	0.081	0.000		0.347	0.000
	Female	1	0.95	1.40	0.94	1.01	1.88	1	1.05	1.74
			0.59–1.53	0.88–2.25	0.55–1.59	0.64–1.58	1.21–2.92		0.77–1.42	1.27–2.37
			0.847	0.159	0.808	0.971	0.005		0.765	0.001
	Male	1	1.28	2.29	1.99	2.09	3.19	1	1.26	2.13
			0.72–2.27	1.18–4.42	0.98–4.01	1.19–3.67	1.74–5.85		0.88–1.82	1.42–3.18
			0.400	0.014	0.056	0.011	0.000		0.205	0.000
Mixed pattern FEV1/FVC < 0.7 and predicted FVC < 80%. (OR, 95% CI, *p*-value)	Total	1	0.39	0.29	1.15	0.37	0.52	1	0.35	0.41
			0.18–0.87	0.10–0.87	0.44–2.97	0.17–0.82	0.24–1.15		0.20–0.59	0.22–0.76
			0.021	0.027	0.771	0.015	0.105		0.000	0.006
	Female	1	0.35	0.25	1.34	0.32	0.45	1	0.27	0.33
			0.11–1.12	0.05–1.39	0.42–4.34	0.09–1.21	0.15–1.36		0.11–0.65	0.13–0.82
			0.077	0.112	0.616	0.093	0.155		0.004	0.017
	Male	1	0.43	0.32	1.03	0.42	0.60	1	0.41	0.48
			0.17–1.12	0.09–1.15	0.27–3.97	0.16–1.10	0.22–1.65		0.20–0.82	0.22–1.07
			0.084	0.079	0.960	0.075	0.319		0.012	0.072
FEV_1_ to predicted FEV_1_ < 80% (OR, 95% CI, *p*-value)	Total	1	0.86	1.17	1.66	1.27	1.67	1	0.83	1.20
			0.58–1.27	0.75–1.80	1.05–2.61	0.88–1.83	1.15–2.43		0.65–1.06	0.92–1.57
			0.443	0.487	0.029	0.205	0.008		0.141	0.187
	Female	1	0.83	1.23	1.44	1.06	1.40	1	0.80	1.14
			0.49–1.40	0.68–2.22	0.68–2.22	0.67–1.69	0.89–2.20		0.57–1.11	0.81–1.59
			0.477	0.493	0.196	0.803	0.144		0.185	0.453
	Male	1	0.99	1.19	2.08	1.62	2.25	1	0.91	1.36
			0.57–1.74	0.63–2.24	1.02–4.21	0.92–2.86	1.23–4.13		0.63–1.32	0.89–2.06
			0.981	0.595	0.043	0.093	0.009		0.625	0.152
FVC to predicted FVC < 80% (OR, 95% CI, *p*-value)	Total	1	0.95	1.56	1.14	1.25	2.05	1	1.00	1.74
			0.68–1.34	1.07–2.28	0.76–1.71	0.91–1.73	1.48–2.84		0.81–1.25	1.36–2.22
			0.785	0.021	0.534	0.172	0.000		0.966	0.000
	Female	1	0.92	1.34	0.97	1.00	1.78	1	0.94	1.64
			0.58–1.43	0.81–2.25	0.57–1.63	0.65–1.54	1.19–2.68		0.69–1.28	1.19–2.25
			0.697	0.256	0.894	0.997	0.006		0.706	0.003
	Male	1	1.16	2.02	1.58	1.75	2.80	1	1.14	2.00
			0.72–1.87	1.17–3.50	0.84–2.96	1.09–2.81	1.67–4.70		0.80–1.61	1.37–2.92
			0.550	0.012	0.154	0.021	0.000		0.471	0.000

Normal weight: BMI = 18.5 kgm^2^–24.4 kgm^2^; overweight: BMI = 25 kgm^2^–29.9 kgm^2^; obesity: BMI ≥ 30 kgm^2^. Metabolically healthy: presence of no metabolic risk factors; metabolically unhealthy: presence of one or more metabolic risk factors. OR = odds ratio, CI = confidence interval, *p*-value = level of significance (<0.05). Adjusted for gender, age, highest education level, mean daily standard alcoholic drinks per day, mean weekly steps, smoking status, diet quality. FEV1 > 80% of predicted = normal. FVC > 80% of predicted = normal.

**Table 4:** Summary of main findings for associations between BMI–metabolic health and all outcomes.

	Restrictive	Obstructive	Mixed	FEV1/pFEV1	FVC/pFVC
MHNW	1	1	1	1	1
MHOW	1.00	**0.39**	**0.39**	0.86	0.95
	0.70–1.47	**0.20–0.77**	**0.18–0.87**	0.58–1.27	0.68–1.34
	0.959	**0.007**	**0.021**	0.443	0.785
MHO	**1.67**	0.36	**0.29**	1.17	**1.56**
	**1.13–2.49**	0.12–1.05	**0.10–0.87**	0.75–1.80	**1.07–2.28**
	**0.011**	0.061	**0.027**	0.487	**0.021**
MUNW	1.23	0.73	1.15	**1.66**	1.14
	0.81–1.86	0.29–1.88	0.44–2.97	**1.05–2.61**	0.76–1.71
	0.333	0.510	0.771	**0.029**	0.534
MUOW	1.37	0.50	**0.37**	1.27	1.25
	0.96–1.95	0.25–1.02	**0.17–0.82**	0.88–1.83	0.91–1.73
	0.081	0.055	**0.015**	0.205	0.172
MUO	**2.23**	**0.33**	0.52	**1.67**	**2.05**
	**1.53–3.25**	**0.14–0.77**	0.24–1.15	**1.15–2.43**	**1.48–2.84**
	**0.001**	**0.011**	0.105	**0.008**	**0.001**

MHNW: metabolically healthy normal weight. MHOW: metabolically healthy overweight. MHO: metabolically healthy obesity. MUNW: metabolically unhealthy normal weight. MUOW: metabolically unhealthy overweight. MUO: metabolically unhealthy obesity. FEV1: forced expiratory volume in 1 s. FVC: forced vital capacity. p FEV1: predicted forced expiratory volume in 1 s. p FVC: predicted forced vital capacity. Significant findings in bold.

## Data Availability

Data are available upon request to the corresponding author.

## Supplementary Materials

The following supporting information can be downloaded at https://www.mdpi.com/article/10.3390/nu16244256/s1: Table S1: Cross-sectional analysis of the associations of WHR–metabolic health group, WHR category on lung function with multiple imputation of missing data; Table S2: Odds of Restrictive lung function pattern according to interaction between each BMI category and metabolic health; Table S3: Odds of Obstructive lung function pattern according to the interaction between BMI category and metabolic health; Table S4: Odds of Mixed lung function pattern ac-cording to the interaction between BMI category and metabolic health; Table S5: Odds of FEV1 to predicted FEV1 < 80% according to the interaction between BMI category and metabolic health; Table S6: Odds of FVC to predicted FVC < 80% according to the interaction between BMI category and metabolic health; Table S7: Odds of Obstructive lung function pattern according to BMI and metabolic health category—results ac-cording to male and female gender with multiple imputation of missing data; Table S8: Odds of Restrictive lung function pattern according to BMI and metabolic health category—results accord-ing to male and female gender with multiple imputation of missing data; Table S9: Odds of Mixed lung function pattern according to BMI and metabolic health cate-gory—results according to male and female gender with multiple imputation of missing data; Table S10: Odds of FEV1 to predicted FEV1 < 80% according to BMI and metabolic health category—results according to male and female gender with multiple imputation of missing data; Table S11: Odds of FVC to predicted FVC < 80% according to BMI and metabolic health category—results according to male and female gender with multiple imputation of missing data; Table S12: Relationship between Metabolic health/Obesity category, BMI category and FEV1, FVC, predicted FEV1, predicted FVC; Table S13: Association between Waist Hip ratio, Metabolic health-Waist Hip ratio category and FEV1, FVC, predicted FEV1, and predicted FVC.

## Abbreviations

ARFS	Australian Recommended Food Score
BMI	Body Mass Index
CI	confidence interval
DAG	directed acyclic graph
IDF	International Diabetes Federation
FEV1	forced expiratory volume after one second
FVC	forced vital capacity
MHNW	metabolically healthy normal weight
MHOW	metabolically healthy overweight
MHO	metabolically healthy obesity
MUNW	metabolically unhealthy normal weight
MUOW	metabolically unhealthy overweight
MUO	metabolically unhealthy obesity
NCEP AT*P*-III	National Cholesterol Education Program Adult Treatment Panel -III report
OR	odds ratio
RERI	Relative Excess Risk due to Interaction
