# Supplementary material for: Metabolic Health, Overweight or Obesity, and Lung Function in Older Australian Adults
Source: Nutrients. 2024 Dec 10;16(24):4256. doi: 10.3390/nu16244256 (PMC11676256; doi:10.3390/nu16244256)
Supplement: Supplementary file 1 [file nutrients-16-04256-s001.zip › nutrients-3327994-supplementary.pdf]

Supplementary information

Supplementary Results for Effect of BMI and metabolic health interaction on lung dysfunction patterns (Results presented in supplementary tables 2, 3,4,5,6)

Restrictive lung function pattern

We investigated how the interaction between BMI and metabolic health is associated with the odds of having a restrictive lung function pattern (see Supplementary Table 2).

For the metabolically unhealthy group, the OR was 1.19 (95% CI: 0.77-1.85, p=0.435). For those who are overweight, the OR was 1.01 (95% CI: 0.70-1.46, p=0.973). Combining overweight and metabolically unhealthy, the OR was 1.39 (95% CI: 0.99-1.95, p=0.054). The reference group consisted of metabolically healthy individuals with normal weight.

Within BMI categories, the OR for restrictive lung function pattern was 1.19 (95% CI: 0.77-1.85, p=0.435) for normal weight and 1.38 (95% CI: 1.04-1.84, p=0.027) for overweight individuals. Within metabolic health groups, the ORs were 1.01 (95% CI: 0.70-1.46, p=0.973) for metabolically healthy overweight and 1.17 (95% CI: 0.81-1.70, p=0.408) for metabolically unhealthy overweight.

The Relative Excess Risk due to Interaction (RERI) for overweight and metabolically unhealthy was -0.59 (95% CI: -2.65 to 1.47, p=0.576), indicating a non-significant negative interaction.

For the metabolically unhealthy individuals, the OR for obesity was 1.68 (95% CI: 1.10-2.56, p=0.017), and for the combination of obesity and metabolic unhealthy, the OR was 2.23 (95% CI: 1.53-3.24, p=0.000). The ORs for restrictive lung function pattern by BMI were 1.19 (95% CI: 0.77-1.85, p=0.435) for normal weight and 1.33 (95% CI: 0.97-1.82, p=0.075) for obesity. In strata of metabolic health, the ORs were 1.68 (95% CI: 1.10-2.56, p=0.017) for metabolically healthy obese individuals and 1.87 (95% CI: 1.28-2.75, p=0.001) for metabolically unhealthy obese individuals.

The RERI for obesity and metabolically unhealthy was -1.18 (95% CI: -4.03 to 1.67, p=0.416), suggesting a non-significant negative interaction.

Obstructive lung function pattern

We investigated the interaction between BMI and metabolic health is associated with the odds of having an obstructive lung function pattern (see Supplementary Table 3).

For the metabolically unhealthy group alone, the odds ratio (OR) was 0.72 (95% CI: 0.29-1.78, p=0.470). For overweight individuals alone, the OR was 0.43 (95% CI: 0.21-0.88, p=0.021).

Combining overweight and metabolically unhealthy, the OR was 0.50 (95% CI: 0.26-0.99, p=0.046). The reference group was metabolically healthy individuals with normal weight.
Within BMI categories, the OR for obstructive lung function pattern was 0.72 (95% CI: 0.29-1.78, p=0.470) for normal weight and 1.17 (95% CI: 0.62-2.19, p=0.628) for overweight individuals. Within metabolic health groups, the ORs were 0.43 (95% CI: 0.21-0.88, p=0.021) for metabolically healthy overweight and 0.70 (95% CI: 0.31-1.60, p=0.389) for metabolically unhealthy overweight.
The RERI for the combination of overweight and metabolic unhealthy was 0.22 (95% CI: -1.21 to 1.65, p=0.762), indicating a potential positive interaction, though not statistically significant. For the metabolically unhealthy group, the OR for obesity was 0.72 (95% CI: 0.29-1.78, p=0.470). For obesity alone, the OR was 0.32 (95% CI: 0.10-0.98, p=0.046), and for the combination of obesity and metabolic unhealthy, the OR was 0.34 (95% CI: 0.14-0.78, p=0.012). The ORs for obstructive lung function by BMI were 0.72 (95% CI: 0.29-1.78, p=0.470) for normal weight and 1.06 (95% CI: 0.33-3.41, p=0.916) for obesity. In strata of metabolic health, ORs were 0.32 (95% CI: 0.10-0.98, p=0.046) for metabolically healthy obese individuals and 0.47 (95% CI: 0.16-1.35, p=0.154) for metabolically unhealthy obese individuals. The RERI for obesity and metabolic unhealthy was -0.49 (95% CI: -2.29 to 1.30, p=0.590), indicating a potential negative interaction, though not statistically significant.

We further analysed how the interaction between BMI and metabolic health is associated with reduced lung function patterns, including mixed patterns, FEV1 to predicted FEV1, and FVC to predicted FVC. See Supplementary Tables 4, 5, and 6.

The results were as follows:

- **Mixed Pattern:** For overweight and metabolically unhealthy individuals, the RERI was 0.33 (95% CI: -0.69 to 1.34, p=0.529), suggesting a positive but not statistically significant interaction. For obesity and metabolically unhealthy individuals, the RERI was 0.24 (95% CI: -0.92 to 1.41, p=0.685), also indicating a positive but not statistically significant interaction.

- **FEV1 to Predicted FEV1:** For overweight and metabolically unhealthy individuals, the RERI was -0.84 (95% CI: -2.60 to 0.91, p=0.347), suggesting a negative but not statistically significant interaction. For obesity and metabolically unhealthy individuals, the RERI was -0.61 (95% CI: -2.40 to 1.18, p=0.501), indicating a negative but not statistically significant interaction.

- **FVC to Predicted FVC:** For overweight and metabolically unhealthy individuals, the RERI was -0.26 (95% CI: -1.54 to 1.02, p=0.693), suggesting a negative but not statistically significant interaction. For obesity and metabolically unhealthy individuals, the RERI was -0.34 (95% CI: -2.16 to 1.48, p=0.714), also indicating a negative but not statistically significant interaction.

Supplementary Tables

Supplementary Table S1. Cross-sectional analysis of the associations of WHR–metabolic health group, WHR category on lung function with multiple imputation of missing data**.**

				Metabolically healthy		Metabolically Unhealthy		Total	WHR category	
**WHR risk level**				**Low risk**	**High risk**	**Low risk**	**High risk**		**Low risk**	**High risk**
Number and Percentage with low and high risk	Female			(454)<br>32.0%	(212)<br>15.0%	(385)<br>27.0%	(387)<br>26.0%	1438	(839)<br>58.0%	(599)<br>42.0%
	Male			(130)<br>10.0%	(470)<br>35.0%	(72)<br>5.0%	(683)<br>50.0%	1355	(202)<br>15.0%	(1,153)<br>85.0%
**Obstructive pattern**<br>FEV1/FVC < 0.7 and FVC ≥ 80% predicted	Female	(570)<br>61%	(368)<br>39%	1	0.36<br>0.09-1.42<br>0.139	1.19<br>0.45-3.17<br>0.715	0.53<br>0.18-1.59<br>0.246	938	1	0.96<br>0.46-1.99<br>0.906
	Male	(125)<br>17%	(614)<br>83%	1	1.25<br>0.29-5.28<br>0.758	0.63<br>0.22-1.85<br>0.393	0.95<br>0.31-2.96<br>0.927	739	1	1.07<br>0.52-2.11<br>0.835
**Restrictive pattern**<br>FEV1/FVC ≥ 0.7 and FVC < 80% predicted	Female	(744)<br>59%	(522)<br>41%	1	0.95<br>0.66-1.37<br>0.786	1.09<br>0.73-1.62<br>0.664	1.36<br>0.98-1.89<br>0.064	1266	1	1.29<br>0.97-1.73<br>0.080
	Male	(161)<br>14%	(975)<br>86%	1	2.29<br>1.07-4.89<br>0.033	1.61<br>0.91-2.85<br>0.099	2.60<br>1.50-4.53<br>0.001	1136	1	1.63<br>1.10-2.41<br>0.016
**Mixed pattern**<br>FEV1/FVC < 0.7 and FVC < 80% predicted	Female	(565)<br>61%	(363)<br>39%	1	1.20<br>0.37-3.88<br>0.753	0.79<br>0.20-3.04<br>0.718	0.80<br>0.28-2.26<br>0.661	928	1	0.97<br>0.36-2.60<br>0.944
	Male	(126)<br>17%	(613)<br>83%	1	1.01<br>0.17-5.99<br>0.994	0.92<br>0.32-2.62<br>0.868	0.84<br>0.35-2.01<br>0.696	739	1	0.73<br>0.29-1.84<br>0.487
**FEV_1 to predicted FEV_1 <80%**	Female	(783)<br>59%	(551)<br>41%	1	1.13<br>0.75-1.70<br>0.565	1.32<br>0.81-2.17<br>0.258	1.53<br>1.08-2.16<br>0.019	1334	1	1.26<br>0.98-1.62<br>0.73
	Male	(180)<br>14%	(1,072)<br>86%	1	2.79<br>1.35-5.77<br>0.007	1.39<br>0.77-2.48<br>0.266	2.47<br>1.43-4.24<br>0.001	1252	1	1.45<br>1.00-2.10<br>0.047
**FVC to predicted. FVC <80%**	Female	(783)<br>59%	(551)<br>41%	1	1.04<br>0.73-1.47<br>0.832	1.14<br>0.76-1.71<br>0.523	1.40<br>1.05-1.88<br>0.023	1334	1	1.36<br>1.00-1.85<br>0.048
	Male	(180)<br>14%	(1,072)<br>86%	1	1.83<br>0.94-3.56<br>0.074	1.39<br>0.78-2.47<br>0.254	2.18<br>1.29-3.70<br>0.005	1252	1	1.26<br>0.87-1.83<br>0.217

Waist Hip Ratio (WHR): Low risk: < 0.85 (females), < 0.90 (males); High risk: ≥0.85 (females), ≥ 0.90 (males). Metabolically healthy: presence of no metabolic risk factor; metabolically unhealthy: presence of one or more metabolic risk factors. OR = odds ratio. CI

= Confidence Interval. *p*-value = level of significance (<0.05). Adjusted for gender, age, highest education level, mean daily standard alcohol drinks per day, mean weekly steps, smoking status, diet quality.

Supplementary Table S2: Odds of Restrictive lung function pattern according to interaction between each BMI category and metabolic health.

BMI category	Metabolically Healthy			Metabolically Unhealthy			OR, CI, p-value within strata of BMI category
	Number with/without outcome	OR, 95%CI, p-value	OR, 95%CI, p-value within strata of metabolically healthy	Number with/without outcome	OR, 95%CI, p-value	OR, 95%CI, p-value within strata of metabolically unhealthy	
18.5 – 24.9	63/211	1	1	52/130	1.19 0.77-1.85 0.435	1	1.19 0.77-1.85 0.435
25 – 29.9	133/362	1.01 0.70-1.46 0.973	1.01 0.70-1.46 0.973	193/374	1.39 0.99-1.95 0.054	1.17 0.81-1.70 0.408	1.38 1.04-1.84 0.027
≥ 30	102/166	1.68 1.10-2.56 0.017	1.68 1.10-2.56 0.017	258/296	2.23 1.53-3.24 0.000	1.87 1.28-2.75 0.001	1.33 0.97-1.82 0.075

BMI = Body Mass Index. Normal weight: BMI = 18.5 kgm^2–24.4 kgm^2; overweight: BMI = 25 kgm^2–29.9 kgm^2; obesity: BMI ≥ 30 kgm^2. Metabolically healthy: presence of no metabolic risk factor; Metabolically unhealthy: presence of one or more metabolic risk factors. OR = odds ratio. CI = Confidence Interval, *p*-value = level of significance (<0.05). Reference group for OR across strata: metabolically healthy normal weight, OR = 1. Reference group for OR within metabolically healthy strata: metabolically healthy normal weight, OR = 1. Reference group for OR within metabolically unhealthy strata: metabolically unhealthy normal weight, OR = 1. OR within strata of BMI category: calculated by dividing OR for metabolically unhealthy group by OR for metabolically healthy group in the same BMI strata. Metabolic health/Overweight: RERI = -0.59, CI: -2.65 – 1.47, *p*-value = 0.576. Metabolic health/Obesity: RERI = -1.18, CI: -4.03 – 1.67, *p*-value = 0.416.

Supplementary Table S3: Odds of Obstructive lung function pattern according to the interaction between BMI category and metabolic health.

BMI category	Metabolically Healthy			Metabolically Unhealthy			OR, CI, p-value within strata of BMI category
	Number with/without outcome	OR, 95%CI, p-value	OR, 95%CI, p-value within strata of metabolically healthy	Number with/without outcome	OR, 95%CI, p-value	OR, 95%CI, p-value within strata of metabolically unhealthy	
18.5 – 24.9	20/211	1	1	11/130	0.72 0.29-1.78 0.470	1	0.72 0.29-1.78 0.470
25 – 29.9	19/362	0.43 0.21-0.88 0.021	0.43 0.21-0.88 0.021	25/374	0.50 0.26-0.99 0.046	0.70 0.31-1.60 0.389	1.17 0.62-2.19 0.628
>= 30	6/166	0.32 0.10-0.98 0.046	0.32 0.10-0.98 0.046	13/296	0.34 0.14-0.78 0.012	0.47 0.16-1.35 0.154	1.06 0.33-3.41 0.916

BMI = Body Mass Index. Normal weight: BMI = 18.5 kgm^2–24.4 kgm^2; overweight: BMI = 25 kgm^2–29.9 kgm^2; obesity: BMI ≥ 30 kgm^2. Metabolically healthy: presence of no metabolic risk factor; Metabolically unhealthy: presence of one or more metabolic risk factors. OR = odds ratio. CI = Confidence Interval. *p*-value = level of significance (<0.05). Reference group for OR across strata: metabolically healthy normal weight, OR = 1. Reference group for OR within metabolically healthy strata: metabolically healthy normal weight, OR = 1. Reference group for OR within metabolically unhealthy strata: metabolically unhealthy normal weight, OR = 1. OR within strata of BMI category: calculated by dividing OR for metabolically unhealthy group by OR for metabolically healthy group in the same BMI strata. Metabolic health/Overweight: RERI = 0.22, CI: -1.21- 1.65, *p*-value = 0.762. Metabolic health/Obesity: RERI = -0.49, CI: -2.29 – 1.30, *p*-value = 0.590.

Supplementary Table S4: Odds of Mixed lung function pattern according to the interaction between BMI category and metabolic health.

BMI category	Metabolically Healthy			Metabolically Unhealthy			OR, CI, p-value within strata of BMI category
	Number with/without outcome	OR, 95%CI, p-value	OR, 95%CI, p-value within strata of metabolically healthy	Number with/without outcome	OR, 95%CI, p-value	OR, 95%CI, p-value within strata of metabolically unhealthy	
18.5 – 24.9	17/211	1	1	11/130	1.14 0.46-2.83 0.782	1	1.14 0.46-2.83 0.782
25 – 29.9	17/362	0.38 0.18-0.81 0.013	0.38 0.18-0.81 0.013	15/374	0.38 0.16-0.94 0.038	0.34 0.14-0.81 0.016	1.02 0.44-2.38 0.965
≥ 30	6/166	0.29 0.11-0.79 0.016	0.29 0.11-0.79 0.016	19/296	0.52 0.24-1.10 0.086	0.46 0.20-1.06 0.068	1.77 0.66-4.76 0.251

BMI = Body Mass Index. Normal weight: BMI = 18.5 kgm^2–24.4 kgm^2; overweight: BMI = 25 kgm^2–29.9 kgm^2; obesity: BMI ≥ 30 kgm^2. Metabolically healthy: presence of no metabolic risk factors. Metabolically unhealthy: presence of one or more metabolic risk factors. OR = odds ratio. CI = Confidence Interval. *p*-value = level of significance (<0.05). Reference group for OR across strata: metabolically healthy normal weight, OR = 1. Reference group for OR within metabolically healthy strata: metabolically healthy normal weight, OR = 1. Reference group for OR within metabolically unhealthy strata: metabolically unhealthy normal weight, OR = 1. OR within strata of BMI category: calculated by dividing OR for metabolically unhealthy group by OR for metabolically healthy group in the same BMI strata. Metabolic health/Overweight: RERI = 0.33, CI: -0.69 – 1.34, *p*-value = 0.529. Metabolic health/Obesity: RERI = 0.24, CI: -0.92 – 1.41, *p*-value = 0.685.

Supplementary Table S5: Odds of FEV1 to predicted FEV1 < 80% according to the interaction between BMI category and metabolic health.

	Metabolically Healthy	Metabolically Unhealthy	OR, CI,

BMI category	Number with/without outcome	OR, 95%CI, p-value	OR, 95%CI, p-value within strata of metabolically healthy	Number with/without outcome	OR, 95%CI, p-value	OR, 95%CI, p-value within strata of metabolically unhealthy	p-value within strata of BMI category
18.5 – 24.9	61/249	1	1	65/138	1.69 1.09-2.63 0.020	1	1.69 1.09-2.63 0.020
25 – 29.9	106/425	0.85 0.59-1.24 0.409	0.85 0.59-1.24 0.409	171/436	1.25 0.86-1.81 0.236	0.74 0.51-1.07 0.105	1.46 1.08-1.98 0.013
>= 30	71/208	1.18 0.77-1.82 0.446	1.18 0.77-1.82 0.446	205/381	1.67 1.15-2.42 0.007	0.99 0.69-1.41 0.945	1.41 1.02-1.97 0.040

BMI = Body Mass Index. Normal weight: BMI = 18.5 kgm^2–24.4 kgm^2; overweight: BMI = 25 kgm^2–29.9 kgm^2; obesity: BMI ≥ 30 kgm^2. Metabolically healthy: presence of no metabolic risk factor. Metabolically unhealthy: presence of one or more metabolic risk factors. OR = odds ratio. CI = Confidence Interval. *p*-value = level of significance (<0.05). Reference group for OR across strata: metabolically healthy normal weight, OR = 1. Reference group for OR within metabolically healthy strata: metabolically healthy normal weight, OR = 1. Reference group for OR within metabolically unhealthy strata: metabolically unhealthy normal weight, OR = 1. OR within strata of BMI category: calculated by dividing OR for metabolically unhealthy group by OR for metabolically healthy group in the same BMI strata. Metabolic health/Overweight: RERI = -0.84, CI: -2.60 – 0.91, *p*-value = 0.347. Metabolic health/Obesity: RERI = - 0.61, CI: - 2.40 – 1.18, *p*-value = 0.501.

Supplementary Table S6: Odds of FVC to predicted FVC < 80% according to the interaction between BMI category and metabolic health.

BMI category	Metabolically Healthy			Metabolically Unhealthy			OR, CI, p-value within strata of BMI category
	Number with/without outcome	OR, 95%CI, p-value	OR, 95%CI, p-value within strata of	Number with/without outcome	OR, 95%CI, p-value	OR, 95%CI, p-value within strata of metabolical	

			metabolically healthy			ly unhealthy	
18.5 – 24.9	80/230	1	1	63/140	1.19 0.79-1.80 0.407	1	1.19 0.79-1.80 0.407
25 – 29.9	150/381	0.96 0.70-1.32 0.800	0.96 0.70-1.32 0.800	208/399	1.24 0.89-1.73 0.198	1.04 0.73-1.49 0.819	1.29 0.99-1.69 0.059
≥ 30	108/171	1.55 1.07-2.23 0.019	1.55 1.07-2.23 0.019	277/309	2.05 1.48-2.85 0.000	1.73 1.22-2.45 0.002	1.33 0.99-1.78 0.060

BMI = Body Mass Index. Normal weight: BMI = 18.5 kgm^2–24.4 kgm^2; overweight: BMI = 25 kgm^2–29.9 kgm^2; obesity: BMI ≥ 30 kgm^2. Metabolically healthy: presence of no metabolic risk factor. Metabolically unhealthy: presence of one or more metabolic risk factors. OR = odds ratio. CI = Confidence Interval. *p*-value = level of significance (<0.05). Reference group for OR across strata: metabolically healthy normal weight, OR = 1. Reference group for OR within metabolically healthy strata: metabolically healthy normal weight, OR = 1. Reference group for OR within metabolically unhealthy strata: metabolically unhealthy normal weight, OR = 1. OR within strata of BMI category: calculated by dividing OR for metabolically unhealthy group by OR for metabolically healthy group in the same BMI strata. Metabolic health/Overweight: RERI = -0.26, CI: -1.54 – 1.02, *p*-value = 0.693. Metabolic health/Obesity: RERI = -0.34, CI: -2.16 – 1.48, *p*-value = 0.714

Supplementary Table S7. Odds of Obstructive lung function pattern according to BMI and metabolic health category—results according to male and female gender with multiple imputation of missing data

BMI category	Male			Female		
	Number with/without outcome	OR, 95%CI, p-value	OR, 95%CI, p-value within male strata	Number with/without outcome	OR, 95%CI, p-value	OR, 95%CI, p-value within female strata
Metabolic Healthy						
18.5-24.9 kgm^2	8/76	1.27 0.47-3.42 0.638	1	12/135	1	1

25-29.9 kgm^2	12/189	0.67<br>0.31-1.46<br>0.314	0.49<br>0.17-1.41<br>0.181	7/173	0.40<br>0.14-1.11<br>0.078	0.40<br>0.14-1.11<br>0.078
≥30 kgm^2	3/66	0.62<br>0.11-3.52<br>0.580	0.33<br>0.05-2.41<br>0.266	3/100	0.31<br>0.08-1.22<br>0.093	0.31<br>0.08-1.22<br>0.093
Metabolically Unhealthy						
18.5-24.9 kgm^2	6/35	2.53<br>1.00-6.36<br>0.49	1.68<br>0.56-5.08<br>0.350	4/95	0.44<br>0.15-1.29<br>0.133	0.44<br>0.15-1.29<br>0.133
25-29.9 kgm^2	20/179	1.35<br>0.63-2.90<br>0.437	0.92<br>0.38-2.20<br>0.841	5/195	0.29<br>0.09-0.94<br>0.039	0.29<br>0.09-0.94<br>0.039
≥30 kgm^2	7/117	0.77<br>0.32-1.87<br>0.556	0.46<br>0.15-1.45<br>0.181	6/179	0.34<br>0.11-1.01<br>0.052	0.34<br>0.11-1.01<br>0.052

Normal weight: BMI = 18.5 kgm^2–24.4 kgm^2; overweight: BMI = 25 kgm^2–29.9 kgm^2; obesity: BMI ≥ 30 kgm^2. Metabolically healthy: presence of no metabolic risk factor; metabolically unhealthy: presence of one or more metabolic risk factors. OR = odds ratio. CI = Confidence Interval. *p*-value = level of significance (<0.05). Reference group for OR females compared with males: males classified as MHNW. Reference group for OR within male strata: males classified as MHNW. Reference group for OR within female strata: females classified as MHNW. MHNW = metabolically healthy normal weight, MHOW = metabolically healthy overweight, MHO = metabolically healthy obesity, MUNW = metabolically unhealthy normal weight, MUOW = metabolically unhealthy overweight, MUO = metabolically unhealthy obesity. Chi2(11) = 25.39. Prob > chi2 = 0.0080 (test for effect modification).

Supplementary Table S8: Odds of Restrictive lung function pattern according to BMI and metabolic health category—results according to male and female gender with multiple imputation of missing data.

Effect Modification (Restrictive Pattern)

BMI category	Male			Female		
	Number with/without outcome	OR, 95%CI, p-value	OR, 95%CI, p-value within male strata	Number with/without outcome	OR, 95%CI, p-value	OR, 95%CI, p-value within female strata
Metabolic Healthy						

18.5-24.9 kgm^2	19/76	0.76 0.42-1.37 0.357	1	44/135	1	1
25-29.9 kgm^2	77/189	1.23 0.82-1.84 0.324	1.56 0.90-2.70 0.113	56/173	0.97 0.63-1.49 0.876	0.97 0.63-1.49 0.876
≥30 kgm^2	54/66	2.55 1.55-4.19 0.000	2.97 1.56-5.67 0.001	48/100	1.39 0.87-2.23 0.164	1.39 0.87-2.23 0.164
Metabolically Unhealthy						
18.5-24.9 kgm^2	27/35	2.29 1.27-4.13 0.006	2.78 1.42-5.45 0.003	25/95	0.77 0.45-1.34 0.358	0.77 0.45-1.34 0.358
25-29.9 kgm^2	129/179	2.16 1.45-3.24 0.000	2.70 1.48-4.92 0.001	64/195	0.98 0.65-1.46 0.906	0.98 0.65-1.46 0.906
≥30 kgm^2	136/117	3.38 2.23-5.12 0.000	3.96 2.21-7.08 0.000	122/179	1.99 1.37-2.89 0.000	1.99 1.37-2.89 0.000

Normal weight: BMI = 18.5 kgm^2–24.4 kgm^2; overweight: BMI = 25 kgm^2–29.9 kgm^2; obesity: BMI ≥ 30 kgm^2. Metabolically healthy: presence of no metabolic risk factor; metabolically unhealthy: presence of one or more metabolic risk factors. OR = odds ratio. CI = Confidence Interval. *p*-value = level of significance (<0.05). Significant *p*-values: MHO (male) OR=2.55, 95% CI: 1.55- 4.19, p-value = 000, MUNW (male) OR = 2.29, 95% CI:1.27-4.13, p-value = 0.006, MUOW (male) OR= 2.16, 95% CI: 1.45- 3.24, p-value = 0.000, MUO (male) OR = 3.38, 95% CI: 2.23-5.12, *p*-value = 0.000, MUO (female) OR = 1.99, 95% CI:1.37-2.89 , *p*-value = 0.000. Reference group for OR females compared with males = males classified as MHNW. Reference group for OR within male strata: males classified as MHNW. Reference group for OR within female strata = females classified as MHNW. MHNW = metabolically healthy normal weight, MHOW = metabolically healthy overweight, MHO = metabolically healthy obesity, MUNW = metabolically unhealthy normal weight, MUOW = metabolically unhealthy overweight, MUO = metabolically unhealthy obesity. Chi2 (11) = 48.38. Prob > chi2 < 0.00001(test for effect modification).

Supplementary Table S9. Odds of Mixed lung function pattern according to BMI and metabolic health category—results according to male and female gender with multiple imputation of missing data.

BMI category	Male			Female		
	Number with/without outcome	OR, 95%CI, p-value	OR, 95%CI, p-value within male strata	Number with/without outcome	OR, 95%CI, p-value	OR, 95%CI, p-value within female strata
Metabolic Healthy						
18.5-24.9 kgm^2	11/76	3.30 1.18-9.18 0.023	1	6/135	1	1
25-29.9 kgm^2	14/189	1.17 0.40-3.43 0.775	0.35 0.15-0.86 0.022	3/173	0.35 0.09-1.42 0.140	0.35 0.09-1.42 0.140
≥30 kgm^2	4/66	0.81 0.17-3.96 0.793	0.25 0.06-1.03 0.054	2/100	0.38 0.07-2.11 0.264	0.38 0.07-2.11 0.264
Metabolically Unhealthy						
18.5-24.9 kgm^2	4/35	2.10 0.33-13.35 0.421	0.64 0.14-2.83 0.544	7/95	1.40 0.42-4.67 0.581	1.40 0.42-4.67 0.581
25-29.9 kgm^2	12/179	1.21 0.45-3.29 0.707	0.37 0.15-0.89 0.027	3/195	0.28 0.06-1.31 0.105	0.28 0.06-1.31 0.105
≥30 kgm^2	13/117	1.60 0.48-5.37 0.441	0.48 0.19-1.25 0.131	6/179	0.45 0.12-1.76 0.246	0.45 0.12-1.76 0.246

Normal weight: BMI = 18.5 kgm^2–24.4 kgm^2; overweight: BMI = 25 kgm^2–29.9 kgm^2; obesity: BMI ≥ 30 kgm^2. Metabolically healthy: presence of no metabolic risk factor; metabolically unhealthy: presence of one or more metabolic risk factors. OR = odds ratio. CI = Confidence Interval. *p*-value = level of significance (<0.05). Significant *p*-values: MHNW (male) OR =3.30, 95% CI: 1.18-9.18, *p*-value = 0.023. Reference group for OR females compared with males: males classified as MHNW. Reference group for OR within male strata: males classified as MHNW. Reference group for OR within female strata: females classified as MHNW. MHNW = metabolically healthy normal weight, MHOW = metabolically healthy overweight, MHO = metabolically healthy obesity, MUNW = metabolically unhealthy normal weight, MUOW = metabolically unhealthy overweight, MUO = metabolically unhealthy obesity. Chi2 (11) = 17.86. Prob > chi2 = 0.0850 (test for effect modification).

Supplementary Table S10. Odds of FEV1 to predicted FEV1<80% according to BMI and metabolic health category—results according to male and female gender with multiple imputation of missing data.

Effect Modification (Fev1 to pFev1>80%)

BMI category	Male			Female		
	Number with/without outcome	OR, 95%CI, p-value	OR, 95%CI, p-value within male strata	Number with/without outcome	OR, 95%CI, p-value	OR, 95%CI, p-value within female strata
Metabolic Healthy						
18.5-24.9 kgm^2	25/89	0.93 0.53-1.65 0.807	1	36/160	1	1
25-29.9 kgm^2	68/224	1.00 0.63-1.56 0.984	1.07 0.64-1.79 0.801	38/201	0.76 0.46-1.25 0.278	0.76 0.46-1.25 0.278
≥30 kgm^2	37/90	1.26 0.73-2.19 0.411	1.35 0.73-2.51 0.337	34/118	1.20 0.72-1.99 0.486	1.20 0.72-1.99 0.486
Metabolically Unhealthy						
18.5-24.9 kgm^2	31/41	2.24 1.25-3.99 0.007	2.40 1.25-4.60 0.008	34/97	1.28 0.77-2.13 0.345	1.28 0.77-2.13 0.345
25-29.9 kgm^2	116/224	1.67 1.08-2.57 0.021	1.79 1.05-3.06 0.033	55/212	0.89 0.57-1.41 0.631	0.89 0.57-1.41 0.631
≥30 kgm^2	121/152	2.39 1.53-3.74 0.000	2.57 1.48-4.45 0.001	84/229	1.15 0.74-1.79 0.537	1.15 0.74-1.79 0.537

Normal weight: BMI = 18.5 kgm^2–24.4 kgm^2; overweight: BMI = 25 kgm^2–29.9 kgm^2; obesity: BMI ≥ 30 kgm^2. Metabolically healthy: presence of no metabolic risk factor; metabolically unhealthy: presence of one or more metabolic risk factors. OR = odds ratio. CI = Confidence Interval. *p*-value = level of significance (<0.05). Significant *p*-values: MUO (male) OR = , *p*-value = . MUO (female) OR = , CI: , *p*-value =. MHNW (female) OR = , CI: , *p*-value = . MUNW (male) OR = 2.24, 95% CI:1.25-3.99, *p*-value = 0.007, MUOW (male) OR = 1.67, 95% CI: 1.08-2.27, p-value = 0.021, MUO (male) OR = 2.39, 95% CI:1.53- 3.74, p-value = 0.000. Reference group for OR females compared with males: males classified as MHNW. Reference group for OR within male strata: males classified as MHNW. Reference group for OR within female strata: females classified as MHNW. MHNW = metabolically healthy normal weight, MHOW = metabolically healthy overweight, MHO = metabolically

healthy obesity, MUNW = metabolically unhealthy normal weight, MUOW = metabolically unhealthy overweight, MUO = metabolically unhealthy obesity. Chi2 (11) = 31.44. Prob > chi2 = 0.0009 (test for effect modification).

Supplementary Table S11. Odds of FVC to predicted FVC<80% according to BMI and metabolic health category—results according to male and female gender with multiple imputation of missing data.

BMI category	Male			Female		
	Number with/without outcome	OR, 95%CI, p-value	OR, 95%CI, p-value within male strata	Number with/without outcome	OR, 95%CI, p-value	OR, 95%CI, p-value within female strata
Metabolic Healthy						
18.5-24.9 kgm^2	30/84	0.94 0.56-1.58 0.814	1	50/146	1	1
25-29.9 kgm^2	91/201	1.16 0.79-1.70 0.441	1.24 0.77-1.99 0.382	59/180	0.91 0.60-1.36 0.634	0.91 0.60-1.36 0.634
≥30 kgm^2	58/69	2.05 1.26-3.34 0.004	2.18 1.25-3.79 0.006	50/102	1.34 0.85-2.10 0.206	1.34 0.85-2.10 0.206
Metabolically Unhealthy						
18.5-24.9 kgm^2	31/41	1.85 1.07-3.21 0.028	1.97 1.07-3.64 0.031	32/99	0.84 0.52-1.37 0.487	0.84 0.52-1.37 0.487
25-29.9 kgm^2	141/199	1.81 1.23-2.66 0.002	1.93 1.19-3.14 0.008	67/200	0.86 0.56-1.32 0.484	0.86 0.56-1.32 0.484
≥30 kgm^2	149/124	2.80 1.85-4.23 0.000	2.98 1.80-4.92 0.000	128/185	1.62 1.11-2.37 0.012	1.62 1.11-2.37 0.012

Normal weight: BMI = 18.5 kgm^2–24.4 kgm^2; overweight: BMI = 25 kgm^2–29.9 kgm^2; obesity: BMI ≥ 30 kgm^2. Metabolically healthy: presence of no metabolic risk factor; metabolically

unhealthy: presence of one or more metabolic risk factors. OR = odds ratio. CI = Confidence Interval. *p*-value = level of significance (<0.05). Significant *p*-values: MHO (male) OR = 2.05, 95% CI: 1.26-3.34, *p*-value = 0.004. MUNW (male) OR = 1.85, CI:1.07-3.21, *p*-value =0.028. MUOW (male) OR = 1.81, CI:1.23-2.66, *p*-value = 0.002. MUO (male) OR = 2.80, CI:1.85-4.23, *p*-value = 0.000. MUO (female) OR= 1.62, 95% CI: 1.11- 2.37, p-value = 0.012. Reference group for OR females compared with males: males classified as MHNW. Reference group for OR within male strata: males classified as MHNW. Reference group for OR within female strata: females classified as MHNW. MHNW = metabolically healthy normal weight, MHOW = metabolically healthy overweight, MHO = metabolically healthy obesity, MUNW = metabolically unhealthy normal weight, MUOW = metabolically unhealthy overweight, MUO = metabolically unhealthy obesity. $Chi^2(11)$ = 43.78. Prob > chi2 <0.00001(test for effect modification).

Supplementary Table S12. Relationship between Metabolic health/Obesity category, BMI category and FEV1, FVC, predicted FEV1, predicted FVC.

Obesity measure		Total				Female				Male			
		Fev1 Coefficient	Fvc Coefficient	pFev1 Coefficient	pFvc Coefficient	Fev1 Coefficient	Fvc Coefficient	pFev1 Coefficient	PFvc Coefficient	Fev1 Coefficient	Fvc Coefficient	pFev1 Coefficient	pFvc Coefficient
Metabolically Healthy	Normal weight 18.5 - 24.9 kgm^2	ref	1	1	1	1	1	1	1	1	1	1	1
	Overweight 25-29.9 kgm^2	0.04<br>-0.02-<br>0.11<br>0.195	-0.03<br>-0.11-<br>0.05<br>0.502	-0.02<br>-0.06-<br>0.02<br>0.281	-0.03<br>-0.08-<br>0.02<br>0.282	0.04<br>-0.04-<br>0.11<br>0.340	-0.02<br>-0.11-<br>0.07<br>0.668	-0.02<br>-0.06-<br>0.02<br>0.395	-0.03<br>-0.89-<br>0.03<br>0.335	0.01<br>-0.09-<br>0.12<br>0.805	-0.09<br>-0.22-<br>0.04<br>0.181	-0.02<br>-0.08-<br>0.04<br>0.446	-0.02<br>-0.10-<br>0.06<br>0.578
	Obesity ≥30 kgm^2	-0.07<br>-0.14-<br>0.01<br>0.099	-0.20<br>-0.29- -<br>0.11<br>0.000	-0.07<br>-0.11- -<br>0.03<br>0.001	-0.08<br>-0.13- -<br>0.02<br>0.008	-0.05<br>-0.14-<br>0.03<br>0.232	-0.17<br>-0.28- -<br>0.07<br>0.001	-0.06<br>-0.11- -<br>0.02<br>0.008	-0.08<br>-0.15- -<br>0.01<br>0.025	-0.10<br>-0.23-<br>0.03<br>0.147	-0.26<br>-0.42- -<br>0.11<br>0.001	-0.08<br>-0.15- -<br>0.00<br>0.040	-0.07<br>-0.17-<br>0.03<br>0.146
Metabolically Unhealthy	Normal weight 18.5 - 24.9 kgm^2	-0.05<br>-0.14-<br>0.04<br>0.258	-0.08<br>-0.19-<br>0.02<br>0.131	-0.00<br>-0.05-<br>0.04<br>0.935	-0.01<br>-0.07-<br>0.06<br>0.854	-0.02<br>-0.12-<br>0.07<br>0.659	-0.04<br>-0.16-<br>0.07<br>0.456	-0.00<br>-0.06-<br>0.05<br>0.873	-0.01<br>-0.09-<br>0.07<br>0.768	-0.12<br>-0.27-<br>0.03<br>0.120	-0.17<br>-0.35-<br>0.01<br>0.063	-0.01<br>-0.09-<br>0.07<br>0.852	-0.01<br>-0.12-<br>0.09<br>0.834
	Overweight 25-29.9 kgm^2	-0.01<br>-0.08-<br>0.06<br>0.768	-0.10<br>-0.18- -<br>0.02<br>0.011	-0.02<br>-0.06-<br>0.01<br>0.202	-0.03<br>-0.08-<br>0.01<br>0.172	0.02<br>-0.06-<br>0.11<br>0.556	-0.06<br>-0.16-<br>0.04<br>0.228	-0.04<br>-0.08-<br>0.00<br>0.057	-0.07<br>-0.13- -<br>0.00<br>0.041	-0.08<br>-0.190-<br>0.03<br>0.148	-0.20<br>-0.32- -<br>0.07<br>0.002	-0.01<br>-0.07-<br>0.05<br>0.794	-0.00<br>-0.08-<br>0.08<br>0.950
	Obesity	-0.11<br>-0.18- -<br>0.04	-0.25<br>-0.33- -<br>0.17	-0.05<br>-0.08- -<br>0.01	-0.06<br>-0.11- -<br>0.01	-0.07<br>-0.15-<br>0.01	-0.17<br>-0.20- -<br>0.08	-0.05<br>-0.09- -<br>0.01	-0.07<br>-0.13- -<br>0.01	-0.20<br>-0.32- -<br>0.08	-0.39<br>-0.53- -<br>0.26	-0.05<br>-0.11-<br>0.01	-0.05<br>-0.13-<br>0.03

	≥30 kgm^2	0.002	0.000	0.008	0.012	0.092	0.000	0.022	0.020	0.001	0.000	0.105	
BMI category	18.5 - 24.9 kgm^2	1	1	1	1	1	1	1	1	1	1	1	1
	25-29.9 kgm^2	0.02<br>-0.03-<br>0.07<br>0.446	-0.05<br>-0.11-<br>0.01<br>0.135	-0.02<br>-0.05-<br>0.00<br>0.116	-0.03<br>-0.07-<br>0.00<br>0.065	0.02<br>-0.04-<br>0.08<br>0.473	-0.04<br>-0.11-<br>0.03<br>0.252	-0.03<br>-0.06- -<br>0.00<br>0.042	-0.05<br>-0.09--<br>0.01<br>0.023	-0.00<br>-0.09-<br>0.09<br>0.964	-0.08<br>-0.19-<br>0.02<br>0.110	-0.01<br>-0.05-<br>0.04<br>0.786	-0.01<br>-0.07-<br>0.05<br>0.696
	≥30 kgm^2	-0.09<br>-0.14--<br>0.03<br>0.005	-0.21<br>-0.28--<br>0.14<br>0.000	-0.05<br>--0.08-<br>-0.02<br>0.000	-0.07<br>-0.11--<br>0.03<br>0.001	-0.07<br>-0.13--<br>0.00<br>0.041	-0.17<br>-0.25--<br>0.10<br>0.000	-0.05<br>-0.09- -<br>0.02<br>0.002	-0.07<br>-0.12--<br>0.03<br>0.002	-0.13<br>-0.23-<br>-0.03<br>0.012	-0.29<br>-0.40--<br>0.17<br>0.000	-0.05<br>-0.09-<br>0.00<br>0.052	-0.06<br>-0.12-<br>0.01<br>0.093

Normal weight: BMI = 18.5 kgm^2–24.9 kgm^2; overweight: BMI = 25 kgm^2–29.9 kgm^2; obesity: BMI ≥ 30 kgm^2. Metabolically Healthy = no risk factor. Metabolically Unhealthy = one or more risk factors. Fev1 (forced expiratory volume in 1 second). Fvc (forced vital capacity). pFev1 (predicted fev1). pFvc (predicted fvc).

Supplementary Table S13. Association between Waist Hip ratio, Metabolic health-Waist Hip ratio category and FEV1, FVC, predicted FEV1, and predicted FVC.

Waist Hip Ratio (WHR)<br><br>Metabolic Health		Female				Male			
		Fev1 Coefficient	Fvc Coefficient	pFev1 Coefficient	pFvc Coefficient	Fev1 Coefficient	Fvc Coefficient	pFev1 Coefficient	pFvc Coefficient
WHR	Low risk	1	1	1	1	1	1	1	1
	High risk	-0.01<br>-0.15-<br>0.12<br>0.852	-0.04<br>-0.22-<br>0.14<br>0.664	0.06<br>-0.10-<br>0.22<br>0.416	0.08<br>-0.13-<br>0.28<br>0.445	0.00<br>-0.16-<br>0.16<br>0.970	-0.03<br>-0.23-<br>0.18<br>0.787	0.07<br>-0.10-<br>0.24<br>0.410	0.09<br>-0.14-<br>0.32<br>0.420
Metabolically Healthy	Low risk	1	1	1	1	1	1	1	1
	High risk	-0.08<br>-0.24-<br>0.08<br>0.302	-0.12<br>-0.32-<br>0.08<br>0.217	-0.08<br>-0.30-<br>0.14<br>0.466	-0.11<br>-0.41-<br>0.18<br>0.422	-0.15<br>-0.42-<br>0.12<br>0.257	-0.15<br>-0.48-<br>0.18<br>0.368	-0.03<br>-0.44-<br>0.39<br>0.890	-0.01<br>-0.33-<br>0.30<br>0.930
Metabolically Unhealthy	Low risk	0.03<br>-0.29-<br>0.35<br>0.828	0.06<br>-0.31-<br>0.44<br>0.725	0.16<br>-0.17-<br>0.49<br>0.332	0.19<br>-0.24-<br>0.63<br>0.357	-0.03<br>-0.25-<br>0.18<br>0.744	-0.05<br>-0.32-<br>0.22<br>0.709	0.03<br>-0.30-<br>0.37<br>0.831	0.03<br>-0.22-<br>0.28<br>0.793

	High risk	-0.11<br>-0.31-0.09<br>0.277	-0.17<br>-0.42-0.08<br>0.174	-0.06<br>-0.31-0.20<br>0.646	-0.09<br>-0.42-0.24<br>0.575	-0.10<br>-0.31-0.11<br>0.340	-0.14<br>-0.41-0.14<br>0.310	0.10<br>-0.20-0.39<br>0.506	0.08<br>-0.15-0.30<br>0.482

WHR = Waist hip ratio (Low risk: < 0.85 in Females, < 0.90 in Males, High risk: ≥ 0.85 in Females, ≥ 0.09 in Males). Metabolically Healthy = no risk factor. Metabolically Unhealthy = one or more risk factors. Fev1 (forced expiratory volume in 1 second). FVC (forced vital capacity). pFEV1 (predicted FEV1). pFVC (predicted FVC).
